# Inhibition of the PI3K/mTOR Pathway in Breast Cancer to Enhance Response to Immune Checkpoint Inhibitors in Breast Cancer

**DOI:** 10.3390/ijms22105207

**Published:** 2021-05-14

**Authors:** Chi Yan, Jinming Yang, Nabil Saleh, Sheau-Chiann Chen, Gregory D. Ayers, Vandana G. Abramson, Ingrid A. Mayer, Ann Richmond

**Affiliations:** 1 Department of Veterans Affairs, Tennessee Valley Healthcare System, Nashville, TN 37212, USA; chi.yan@vanderbilt.edu (C.Y.); jinming.yang@vanderbilt.edu (J.Y.); nabil.saleh@vanderbilt.edu (N.S.); 2 Department of Pharmacology, Vanderbilt University School of Medicine, Nashville, TN 37240, USA; 3 Department of Biostatistics, Vanderbilt University Medical Center, Nashville, TN 37232, USA; sheau-chiann.chen.1@vumc.org (S.-C.C.); dan.ayers@vumc.org (G.D.A.); 4 Department of Medicine, Vanderbilt University Medical Center, Nashville, TN 37232, USA; vandana.abramson@vumc.org (V.G.A.); ingrid.mayer@vumc.org (I.A.M.)

**Keywords:** PI3K/mTOR, breast cancer, immune checkpoint inhibitor, cytotoxic T cells

## Abstract

Objectives: Inhibition of the PI3K/mTOR pathway suppresses breast cancer (BC) growth, enhances anti-tumor immune responses, and works synergistically with immune checkpoint inhibitors (ICI). The objective here was to identify a subclass of PI3K inhibitors that, when combined with paclitaxel, is effective in enhancing response to ICI. Methods: C57BL/6 mice were orthotopically implanted with syngeneic luminal/triple-negative-like PyMT cells exhibiting high endogenous PI3K activity. Tumor growth in response to treatment with anti-PD-1 + anti-CTLA-4 (ICI), paclitaxel (PTX), and either the PI3Kα-specific inhibitor alpelisib, the pan-PI3K inhibitor copanlisib, or the broad spectrum PI3K/mTOR inhibitor gedatolisib was evaluated in reference to monotherapy or combinations of these therapies. Effects of these therapeutics on intratumoral immune populations were determined by multicolor FACS. Results: Treatment with alpelisib + PTX inhibited PyMT tumor growth and increased tumor-infiltrating granulocytes but did not significantly affect the number of tumor-infiltrating CD8^+^ T cells and did not synergize with ICI. Copanlisib + PTX + ICI significantly inhibited PyMT growth and increased activation of intratumoral CD8^+^ T cells as compared to ICI alone, yet did not inhibit tumor growth more than ICI alone. In contrast, gedatolisib + ICI resulted in significantly greater inhibition of tumor growth compared to ICI alone and induced durable dendritic-cell, CD8^+^ T-cell, and NK-cell responses. Adding PTX to this regimen yielded complete regression in 60% of tumors. Conclusion: PI3K/mTOR inhibition plus PTX heightens response to ICI and may provide a viable therapeutic approach for treatment of metastatic BC.

## 1. Introduction

The phosphatidylinositol 3-kinase/protein kinase B/mammalian target of rapamycin (PI3K/mTOR) pathway is frequently altered in breast cancer (BC) [1]. The oncogenic activation of PI3K/mTOR may occur through the mutation or overexpression of upstream regulators (e.g., epidermal growth factor receptor [EGFR, 4%]; NRAS, 3%; KRAS, 4%), amplification or activating mutations in the PI3K catalytic subunit α (PI3KCA) (47%), loss of function or expression of phosphatase and tensin homolog (PTEN) (6%), or amplification of AKT (also known as protein kinase B, 5%) (cBio-TCGA). PI3Ks are heterodimers and exist in four isoforms, namely PI3Kα, β, γ, δ. These isoforms convert phosphatidylinositol-4,5-bisphosphate (PIP2) into phosphatidylinositol-3,4,5-trisphosphate (PIP3), which in turn controls a range of cellular actions including cell growth, migration, metabolism, survival, and proliferation [2]. PI3Kαβ are expressed ubiquitously and are often constitutively active in BC, while PI3Kγδ are predominately expressed in leukocytes, suggesting that, in addition to reprogramming tumor immune microenvironment, targeting PI3Ks will also have direct effect on breast cancer cells. However, extensive grade 3 and 4 toxicity present a major limitation of continuous inhibition of PI3Ks in patients [3], and new therapeutic combinations for PI3K inhibition in BC are needed.

The discovery of immune checkpoint inhibitors (ICIs) targeting cell death protein (PD)-1/PD-Ligand 1 (PD-L1) and cytotoxic T-lymphocyte-associated protein (CTLA)-4 is revolutionizing cancer treatments [4]. ICIs unleash anti-tumor immune responses, including CD8^+^ and CD4^+^ T cell, dendritic cell (DC), and macrophage effector function through blocking negative immune checkpoint proteins [4,5]. However, breast tumors are often unable to respond to ICIs because they are “immunologically cold”, in part due to a relatively low mutational load, and many of these tumors feature an immune-suppressive tumor microenvironment (TME) [6]. Nevertheless, combining ICI therapy with the microtubule poison paclitaxel (PTX) induces immunogenic tumor cell death and enhances anti-cancer immunity [7]. Recently, nanoparticle albumin-bound (nab)-PTX combined with anti-PD-L1 antibody atezolizumab was FDA approved for treatment of locally advanced, unresectable TNBC based on the finding of when patients with PD-L1-positive tumors were treated with this combination of therapy, there was an improvement in overall survival [8]. Furthermore, pre-clinical studies using combined treatment with PTX and either a β-isoform-sparing PI3K inhibitor taselisib or pan-AKT inhibitor ipatasertib reduced proliferative and metastatic effects of breast cancer cells, as compared to either therapeutic strategy alone [9]. We have shown in mouse models that BKM120, a pan-PI3K inhibitor, slows BC tumor growth and reduces metastasis, enhances antitumor immunity, and increases sensitivity to ICI [10]. However, severe toxicity of BKM120 was observed in patients due to the ability of BKM120 to cross the blood-brain barrier and induce severe depression, which resulted in withdrawal of the drug [11]. A phase II clinical trial study of combining PTX with mTOR inhibitor everolimus showed that while PTX is well tolerated, the combination treatment was associated with more adverse events without improvement in pathologic complete response or clinical response in patients with stage II/III triple-negative breast cancer (TNBC) [12]. Subsequently, clinical trials turned to examine effects of either isoform-specific or dual PI3K/mTOR inhibitors in patients with advanced or metastatic BC. To date, results from trials combining PI3Kα or PI3K/mTOR inhibition with ICI therapy have not been reported. The objective of this study was to evaluate the effects of isotype-specific PI3Kα inhibitors versus alternative pan-PI3K inhibitors or PI3K/mTOR pathway inhibitors in combination with PTX and ICI for treatment of BC.

Herein, we show that combined treatment with a PI3Kα isoform-specific inhibitor, alpelisib, and PTX induces beneficial, but not durable, anti-tumor granulocyte responses in BC patient peripheral blood. Using the polyomavirus middle T (PyMT) murine breast cancer model, we validated the alpelisib induced anti-tumor granulocyte responses. However, the addition of ICI to this therapeutic regimen did not enhance response to alpelisib + PTX therapy. The pan-PI3K inhibitor, copanlisib, combined with ICI resulted in partial remission of PyMT breast tumor growth, which was associated with an elevation of CD69^+^CD8^+^ activated cytotoxic T cells in the TME. We further showed that when the PI3K/mTOR dual inhibitor, gedatolisib, was combined with ICI therapy, there was an ~85% inhibition of growth of PyMT breast tumors. This combinatorial therapy induced durable DC, T-cell and natural killer (NK)-cell responses both systemically and locally in the TME. Notably, the addition of PTX to the gedatolisib + ICI regimen achieved the best response, with complete regression of 60% of PyMT tumors after 30 days of treatment.

Analysis of a BC patient dataset from the Single Cell RNA-Seq Expression Atlas suggest that while many PI3K/mTOR-associated genes (e.g., *PI3KCA*, *PI3KCB*, *MTOR*, *RSP6,* and *4E-BP1*) are ubiquitously expressed, *PI3KCD* (encoded for PI3Kδ) and *PIK3CG* (encoded for PI3Kγ) are preferentially expressed in PD-1^+^ lymphocytes in the primary and metastatic TNBC. Therefore, the combination therapy of gedatolisib + PTX + ICI may translate into successful clinical outcome in BC patients, especially TNBC, and warrants further evaluation.

## 2. Results

### 2.1. Combination of PI3Kα Isoform-Specific Inhibitor, Alpelisib, and Paclitaxel Induces Granulocyte Responses but Does Not Synergizes with Immunotherapy

**Figure 1:**
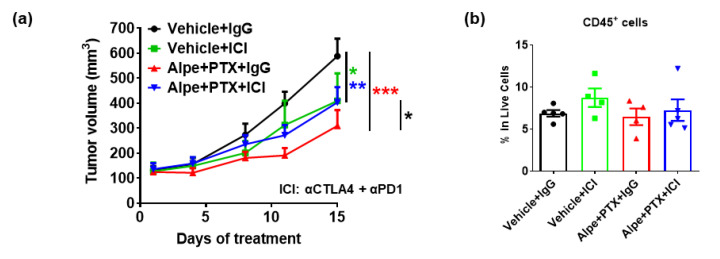

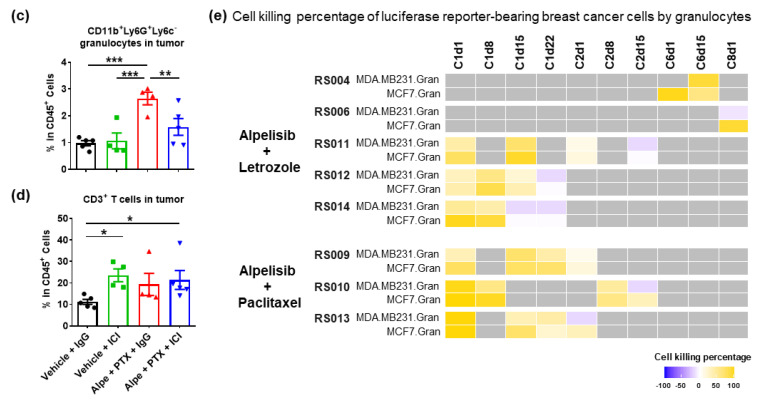
Combination of PI3Kα isoform-specific inhibitor, alpelisib, and paclitaxel induces granulocyte responses but did not synergize with immunotherapy. Female C57BL/6 mice were injected with 500,000 PyMT cells in mammary fat pads, and treatment with 50 mg/kg alpelisib (5 days/week, oral), 10 mg/kg PTX (every 3 days, retroorbital), and ICI (200 ug/mouse anti-PD-1 and 100 ug/mouse anti-CTLA-4 every 3 days, intraperitoneal) was started when tumors reached 125 mm^3^. (**a**–**d**) Primary PyMT tumor samples from mice in each experimental group were collected and immune profiled by flow cytometric analyses (n = 5 per group). (**e**) Granulocytes (Gran) were isolated and collected by density gradient centrifugation from 250 ul of peripheral blood of patients and co-cultured with breast cancer cells MDA-MB231 and MCF7. A heatmap of percent killing of cancer cells is shown. Data not available are shown in gray cells. C, cycle of treatment. d, days of treatment in the cycle. Where indicated, * *p* < 0.05; ** *p* < 0.01; *** *p* < 0.001.

The PI3Kα inhibitor, alpelisib, has shown some promise for treatment of ER^+^ breast cancer in combination with fulvestrant [13]. In addition, alpelisib combined with CDK4/6 inhibition enhances response to ICI in pRb^+^ triple-negative BCs [14]. Since PI3Kα is frequently mutated or activated in BCs, we chose to investigate the effects of alpelisib combined with PTX in response to ICI (anti-PD-1 and anti-CTLA-4) using a luminal, TNBC-like PyMT orthotopic implantation mouse model of breast cancer in C57BL/6 mice. Treatment of PYMT tumor-bearing mice with (1) vehicle for alpelisib and vehicle for PTX + IgG, (2) alpelisib (50 mg/kg) + PTX (10 mg/kg) + IgG, (3) vehicles + ICI (200 ug anti-PD-1 and 100 ug anti-CTLA-4), or (4) alpelisib + PTX + ICI was initiated when the tumors were ~125 mm^3^, and continued for 2 weeks. After 2 weeks of treatment, there was a significant reduction in tumor volume of mice treated with vehicles + ICI (*p* = 0.02), alpelisib + PTX + IgG (*p* < 0.001), and alpelisib + PTX + ICI (*p* = 0.004) compared to vehicles + IgG-treated mice (Figure 1a). In addition, a significant reduction in tumor volume was observed in mice treated with alpelisib + PTX + IgG compared to ICI + vehicle-treated mice (*p* = 0.05). However, there was no significant difference in the tumor volume of mice treated with alpelisib + PTX + ICI versus alpelisib + PTX + IgG or with vehicles + ICI, suggesting that the addition of ICI to PI3Kα inhibitor plus PTX did not improve the response to ICI alone in mice bearing PyMT tumors.

To obtain more insight into the immune responses in the tumor microenvironment (TME), multicolor fluorescence-activated cell sorting (FACS) analysis was performed on each tumor to characterize and quantitate the tumor immune infiltrate. There was no significant difference in total CD45^+^ leukocyte abundance in the tumors across all treatment groups (Figure 1b), where the gating strategy of immune composition in the TME is shown in Figure 1c. We observed a significant increase in the frequency of CD11b^+^Ly6G^+^Ly6c^lo^ granulocytes in tumors of mice treated with alpelisib + PTX + IgG compared to vehicle + IgG (*p* < 0.001) and vehicle + ICI-treated (*p* = 0.002) mice (Figure 1d). The addition of ICI to this alpelisib + PTX therapeutic regimen significantly (*p* = 0.01) reduced granulocyte frequency to the levels as in vehicle + IgG-treated tumors. Furthermore, there was a significant increase in T cells of tumors from mice treated with vehicle + ICI (*p* = 0.04) or with alpelisib + PTX + ICI (*p* = 0.01), but not alpelisib + PTX, as compared to vehicle + IgG-treated mice (Figure 1e). However, the addition of alpelisib + PTX to ICI therapy did not further promote a T cell-response in the TME. There were no significant changes in monocytes, macrophages, NK cells, total DC, and T regulatory cells (Tregs) across treatment groups (Supplementary Figure S1). Together, while mice treated with alpelisib + PTX did show inhibition of tumor growth with induced granulocyte responses in the TME, alpelisib + PTX did not increase the sensitivity of PyMT tumors to ICI therapy.

### 2.2. Alpelisib Induces Beneficial, but Not Durable, Granulocyte Responses in Breast Cancer Patients

Evaluation of the leukocyte profile in peripheral blood of patients enrolled in clinical trials (NCT01791478, NCT01872260, or NCT02379247) combining PI3K antagonists alpelisib with letrozole or PTX in our previous work showed that an early cytotoxic T-cell response (CD45^+^CD8^+^CD107a^+^) over cycle 1 of treatment significantly correlated with the duration of therapeutic response (months), suggesting that a patient’s early cytotoxic T-cell response from peripheral blood could serve as a predictive biomarker for therapeutic response to PI3K inhibition [15]. Given that alpelisib + PTX increased the frequency of granulocytes, but not T cells, in the PyMT tumors, to determine whether our findings regarding alpelisib induction of granulocytes observed in mouse PyMT BC model are recapitulated in human BCs (Figure 1e and Supplementary Figure S2), we further evaluated the granulocyte response on these clinical trials of alpelisib (PI3Kα inhibitor) plus letrozole (RS-004, RS-006, RS-011, RS-012 and RS-014) or paclitaxel (RS-009, RS-010 and RS-013), for a period of up to 8 cycles or until withdrawal from the trial. Reasons for going off-trial were depression, progressive disease, or death. While there was no significant alteration of total CD45^+^ leukocytes in the peripheral blood after the first cycle of treatment (Supplementary Figure S2a), BC patients who responded to treatment with a positive fold-change in their granulocyte population had an average duration of treatment response of 49.2 ± 11.9 months (Supplementary Figure S2b). In contrast, BC patients who responded to treatment with a negative fold-change in their granulocyte population had an average duration of therapeutic response of only 20.25 ± 14.9 months. Using multicolor FACS analysis, we showed that there is a positive trend of correlation (correlation coefficient = 0.80, *p* = 0.13 on cycle 1 day 8; correlation coefficient = 0.50, *p* = 0.45 on cycle 1 day 15) between the early granulocyte (CD45^+^CD66b^+^) population relative to progression free survival (PFS) (months) over cycle 1 of treatment (Supplementary Figure S2c). Furthermore, we isolated granulocytes from patient peripheral blood leukocytes (PBL) and evaluated the functional ability of these granulocytes to kill human ER^+^ MCF7 and triple-negative MDA-MB231 breast cancer cells ex vivo over the cycles of therapy. Our ex vivo killing assay suggested an effective 60–100% killing of tumor cells by PBL-sorted granulocytes from peripheral blood of alpelisib plus letrozole or paclitaxel-treated patients over cycle 1 of treatment (Figure 1f). While the PBL-sorted granulocytes of cycle 1–2 were not available for 2/5 patients with alpelisib+letrozole treatment (RS004 and RS006), when we analyzed the PBL-sorted granulocytes from alpelisib + letrozole-treated patients (RS-011, RS-012 and RS-014) and the alpelisib + paclitaxel-treated patients (RS-009, RS-010 and RS-013), we observed these granulocytes gradually lost their anti-tumor effects on tumor cell growth inhibition in the late cycle 1 to cycle 2 of treatments, suggesting that while the alpelisib-induced granulocyte-mediated anti-tumor effects may have potential benefits, the anti-tumor activity of these granulocytes is not maintained.

### 2.3. Pan-PI3K Inhibitor, Copanlisib, Plus Immune Therapy Produced Partial Remission of PyMT Breast Tumor Growth

**Figure 2:**
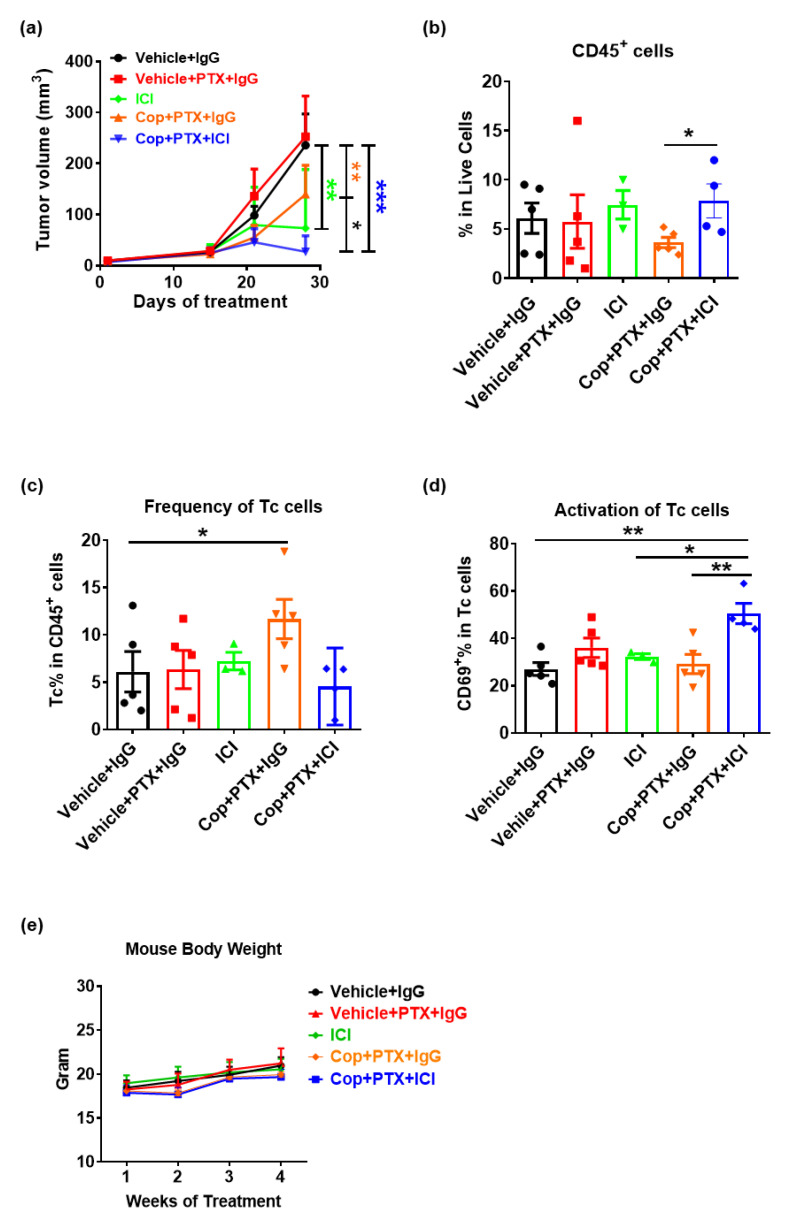
Pan-PI3K inhibitor, copanlisib, plus immune therapy produced partial remission of PyMT breast tumor growth. Female C57BL/6 mice were injected with 100,000 PyMT cells in the 4^th^ mammary fat pads. (**a**) Tumor measurements were recorded throughout the course of treatment to produce tumor growth curve, and treatments with 0.8 mg/kg copanlisib (every 2 days, intravenous), 10 mg/kg PTX (every 3 days, retroorbital), and ICI (200 ug anti-PD-1 and 100 ug anti-CTLA-4 every 3 days, intraperitoneal) were started 1 week post tumor cell implantation. Tumor samples were collected from each treatment group at day 28 of treatment and immune profiled by FACS analysis to measure (**b**) all immune cells and (**c**,**d**) immune cell subpopulations in the tumor. (**e**) Mouse body weight recorded for 28 days of treatment. (All graphs show mean ± SEM n ≥ 4 per group.) Where indicated, * *p* < 0.05; ** *p* < 0.01.

Given that the combination of PI3Kα-specific inhibition plus PTX failed to sensitize PyMT tumors for ICI therapy, we postulated that it might be necessary to also inhibit other PI3K isoforms (e.g., PI3Kγ) based on the findings that PI3Kγ or pan-PI3K inhibition can shift the tumor immune microenvironment to one that is anti-tumor by directly affecting macrophage phenotype [10,16]. As noted earlier, severe toxicity issues coupled with poor clinical response resulted in withdrawal of the pan-PI3K inhibitor, BKM120, from the clinic [10,11]. However, another pan-PI3K inhibitor, copanlisib, has been FDA approved for treatment of relapsed follicular lymphoma, and its toxicity effects are tolerable [17]. Therefore, we sought to determine whether combined treatment with copanlisib + PTX plus ICI would be effective for treatment of PyMT breast cancer in the preclinical model. As shown in Figure 2a, after 3 weeks of treatment, there were significant decreases in tumor volume of mice treated with copanlisib + PTX + IgG (*p* = 0.001), vehicle + ICI (*p* = 0.001), and copanlisib + PTX + ICI (*p* < 0.001) compared to vehicle + IgG-treated mice. In addition, there was a significant decrease in tumor volume of mice treated with copanlisib + PTX + ICI versus copanlisib + PTX + IgG-treated (*p* = 0.02) mice, suggesting that the addition of ICI enhanced response to copanlisib + PTX; however, the addition of copalisib + PTX did not enhance the response to ICI alone.

The immune infiltrate of each tumor was determined by multicolor FACS analysis. While there were no significant differences in total CD45^+^ tumor-infiltrated leukocyte (TIL) abundance in tumors across all treatment groups compared to vehicle + IgG, the addition of ICI into copanlisib + PTX increased the frequency of TIL in the TME (*p* = 0.04) (Figure 2b). Further characterization of immune cell subpopulations indicated a significant increase in the CD8^+^ cytotoxic T cell (Tc) content in tumors of mice treated with copanlisib + PTX + IgG (*p* = 0.02) as compared to tumors from mice treated with vehicle + IgG (Figure 2c). In addition, copanlisib + PTX + ICI increased the abundance of activated (CD69^+^) Tc cells as compared to tumors from mice treated with vehicle + IgG (*p* = 0.002), vehicle + ICI (*p* = 0.048), or copanlisib + PTX + IgG (*p* = 0.006) (Figure 2d). There were no significant changes in granulocyte, monocyte, macrophage, or natural killer cell content of tumors in response to treatments. These data suggest that pan-PI3K inhibition by copanlisib, combined with PTX, enhanced Tc cell responses and promoted the activation of Tc cells that enhanced the response of BC to ICI therapy. Therefore, as we demonstrated previously with BKM-120 [10], pan-inhibition of the PI3K pathway may indeed be effective in enhancing response to ICI therapy. However, the effectiveness of this combination, while tolerable and without loss of mouse body weight (Figure 2e), is limited to a partial remission. mTOR is another signal transducer that acts downstream of PI3K activation. We postulated that an inhibitor that inhibited both mTOR and PI3Ks might be more effective that PI3K inhibition alone when combined with ICI therapy for breast cancer treatment.

### 2.4. Dual PI3K/mTOR Inhibitor, Gedatolisib, Plus Immune Therapy Effectively Stopped PyMT Breast Tumor Growth

**Figure 3:**
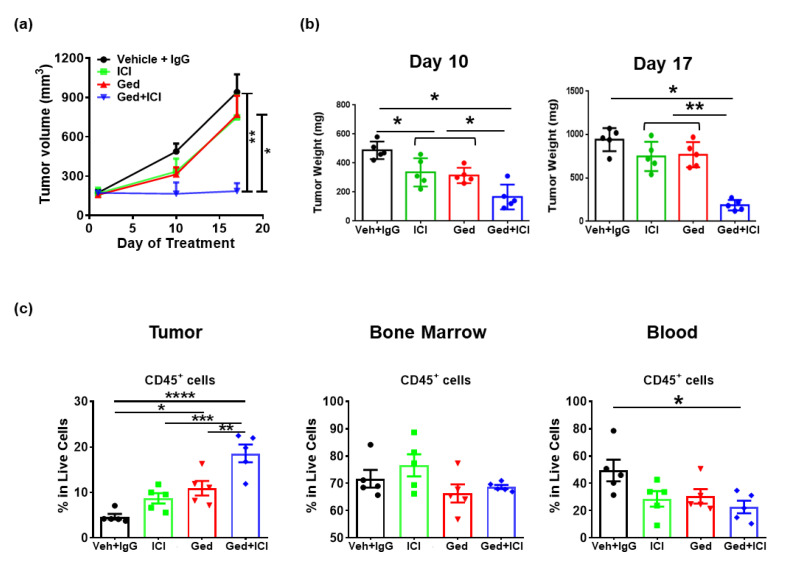
PI3K/mTOR inhibitor, gedatolisib, plus immune therapy completely restrained PyMT breast tumor growth. (**a**–**c**) Female C57BL/6 mice were injected with 500,000 PyMT cells in the 4th mammary fat pad. Mice were treated with immune therapy (CTLA4 at 100 ug/mouse and PD-1 Abs at 200 ug/mouse every 3 days, intraperitoneal) and/or gedatolisib (12 mg/kg, twice a week, intravenous), and vehicle plus isotype IgG antibodies were treated as control. Treatments were started when tumors reached 150 mm^3^. Tumor weights were measured at days 10 and 17. Primary tumor, bone marrow (BM) and lung samples were collected at day 10 post treatment and immune profiled by flow cytometric analyses (n = 5 per group). Where indicated, * *p* < 0.05; ** *p* < 0.01; *** *p* < 0.001; and **** *p* < 0.0001.

Gedatolisib is a potent and reversible inhibitor of PI3Ks and mTOR. IC50 values for PI3Kα, PI3Kβ, PI3Kδ, PI3Kγ, and mTOR are 0.4 nmol/L, 6 nmol/L, 8 nmol/L, 6 nmol/L, and 10 nmol/L, respectively [18]. To develop mechanistic insight into how gedatolisib might enhance sensitivity to ICI therapy in preclinical models, PyMT cells (1 × 10^5^) were injected into the fourth mammary fat pad equivalent on either flank of 6–8-week-old female C57BL/6 mice. Treatments were as follows: (1) vehicle for gedatolisib + IgG (300 μg), (2) gedatolisib (12 mg/kg) + IgG (300 μg), (3) vehicle for gedatolisib + ICI (200 ug anti-PD-1 and 100 ug anti-CTLA-4), or (4) gedatolisib (12 mg/kg) + ICI (200 ug anti-PD-1 and 100 ug anti-CTLA-4). Treatments were initiated when the tumors were ~150 mm^3^ and continued for 17 days. We sampled the tumors at early (day 10 post-treatment) and late (day 17 post-treatment) stages. There was a significant reduction in tumor volume in mice treated with gedatolisib + ICI (*p* = 0.005) compared to vehicle + IgG-treated mice, while no significant change of tumor volume was observed in monotherapy treatments (Figure 3a). Importantly, there was a significant reduction in tumor volume of mice treated with gedatolisib + ICI compared to tumors from mice treated with vehicle + gedatolisib (*p* = 0.025) or vehicle + ICI (*p* = 0.020). Consistent with the tumor volume results, gedatolisib + ICI reduced the tumor weight at day 10 and 17 compared to the tumors from mice treated with vehicle + IgG or monotherapy (Figure 3b). Notably, gedatolisib + ICI completely stopped tumor growth for the entire treatment period with ~85% growth inhibition compared to the vehicle + IgG-treated tumors.

Immune profiling of local (TME) and systemic (bone marrow [BM] and peripheral blood) responses were conducted by multi-color FACS analysis (Figure 3c). Treatment of gedatolisib + IgG (*p* = 0.03) resulted in a significant increase in the CD45^+^ TIL average frequency from 4.7% to 10.94% in all live cells in the TME compared to vehicle + IgG treatment. Importantly, the combination of gedatolisib + ICI further increased the abundance of CD45^+^ TIL to 18.61% compared to vehicle + ICI (8.7%) (*p* = 0.0008), gedatolisib + IgG (10.94%) (*p* = 0.007) or vehicle + IgG (4.7%) (*p* < 0.0001) treatments. There were no significant changes in the abundance of CD45^+^ leukocytes in the BM, while a ~30% reduction was observed in the peripheral blood in the mice treated with gedatolisib + ICI (*p* = 0.03) compared to vehicle + IgG. These results suggest that the gedatolisib + ICI-induced immune cell infiltration into tumors likely resulted from the increased recruitment of leukocytes from blood circulation into the TME, and did not result from *de novo* hematopoiesis in the BM.

**Figure 4:**
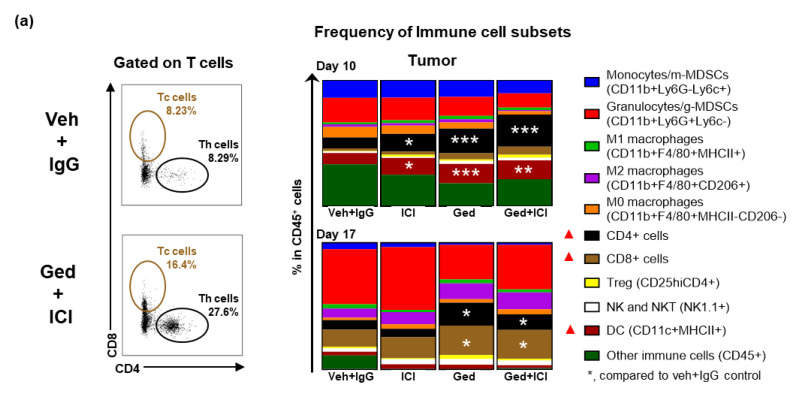

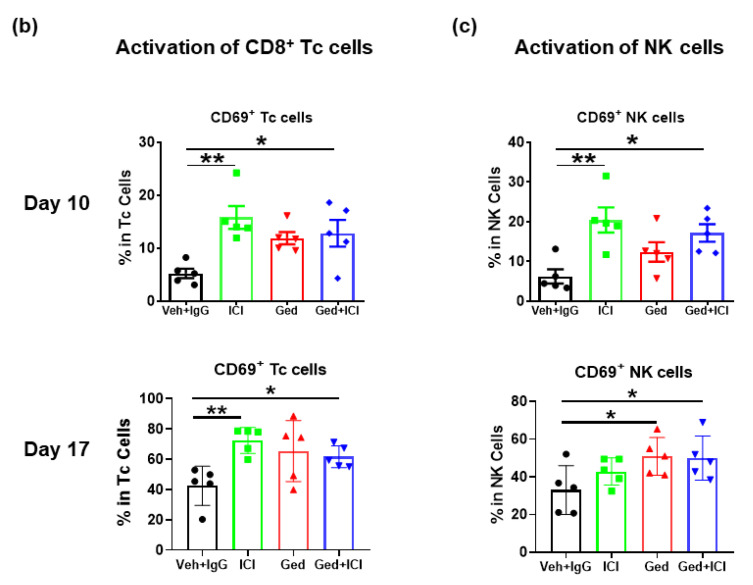
Gedatolisib + Immune induces durable local DC, T-cell and NK-cell responses. (**a**–**c**) Female C57BL/6 mice were injected with 500,000 PyMT cells in fat pad. Mice were treated with immune therapy (CTLA4 at 100 ug/mouse and PD-1 Abs at 200 ug/mouse every 3 days, intraperitoneal) and/or gedatolisib (12 mg/kg, twice a week, intravenous), and vehicle plus isotype IgG antibodies were treated as control. Treatments were started when tumors reached 150 mm^3^. Immune profiling of primary tumor samples was analyzed by flow cytometry (n = 5 per group). Where indicated, * *p* < 0.05; ** *p* < 0.01.

FACS analyses were performed to characterize immune cell subpopulations in tumors at early (10 days) and late (17 days) treatment stages (Figure 4a). At the early treatment timepoint, there was a significant induction of CD4^+^ T cell content in the tumors with treatments of vehicle + ICI (13.7%, *p* = 0.01), gedatolisib + IgG (19.6%, *p* = 0.0002), or gedatolisib + ICI (25.5%, *p* < 0.0001), compared to vehicle + IgG (8.6%). While vehicle + ICI (6.9%, *p* = 0.743) did not maintain the increased level of CD4^+^ T cells at the late treatment timepoint, gedatolisib + IgG (19.2%, *p* = 0.014) or gedatolisib + ICI (12.4%, *p* = 0.041) resulted in a durable CD4^+^ T-cell response as observed in the late treatment timepoint, compared to vehicle + IgG (7.4%). In addition, there was a significant induction of CD8^+^ T cells at the late treatment timepoint in the tumors treated with gedatolisib + IgG (24.5%, *p* = 0.02) or gedatolisib + ICI (22.8%, *p* = 0.05) compared to vehicle + IgG (13.6%). Accompanied with the treatment-induced T-cell responses, the frequency of DCs in the TME was promoted at the early timepoint of treatments with vehicle + ICI (13.14%, *p* = 0.02), gedatolisib + IgG (15.36%, *p* = 0.0002), or gedatolisib + ICI (14.88%, *p* < 0.0001), compared to vehicle + IgG (9.0%). Besides increased frequency of T cells and DCs in the TME, gedatolisib + ICI treatment also induced a 1.5–2-fold increase in the activation (CD69^+^) of CD8^+^ Tc cells (Figure 4b, *p* = 0.0219 at day 10, *p* = 0.0198 at day 17) and NK cells (Figure 4c, *p* = 0.0050 at day 10, *p* = 0.0308 at day 17) compared to vehicle + IgG at both early and late treatment timepoints. There was a clear induction of granulocytic population and M2 macrophages in the tumors at day 17 compared to day 10, which was independent of therapy types and could be a potential mechanism of resistance in the residual tumors.

Next, we conducted multicolor FACS analysis on systemic immune subpopulations in the BM and peripheral blood (Supplementary Figure S3a). Consistent with the TME immune profiling results, there was a significant increase in CD8^+^ Tc cells in the BM of mice treated with gedatolisib + ICI (early timepoint, from 5.2% to 11.6%; late timepoint, from 5.7% to 17.0%), but not gedatolisib monotherapy, compared to vehicle + IgG. Interestingly, the gedatolisib + ICI-induced CD8^+^ Tc-cell response is associated with a reduction in granulocytes in the BM. In the blood circulation, there were significant reductions in granulocytes at early treatment timepoint and an induction of monocytes at the late treatment timepoint across all treatment groups compared to vehicle + IgG. Notably, gedatolisib + IgG (15%) and gedatolisib + ICI (20%) resulted in a 1.5-fold and 2-fold increase in circulating MHCII^+^CD11c^+^ DCs in the peripheral blood compared to the vehicle + IgG (5%) at the early (Supplementary Figure S3b), but not late (Supplementary Figure S3c), treatment timepoints. Taken together, these results suggested that the combination of gedatolisib and ICI induces durable local and systemic DC and T-cell immune responses in PyMT breast cancer tumors.

### 2.5. Gedatolisib Plus PTX and Immune Therapy Resulted in 60% Complete Regression of PyMT Breast Tumors

**Figure 5:**
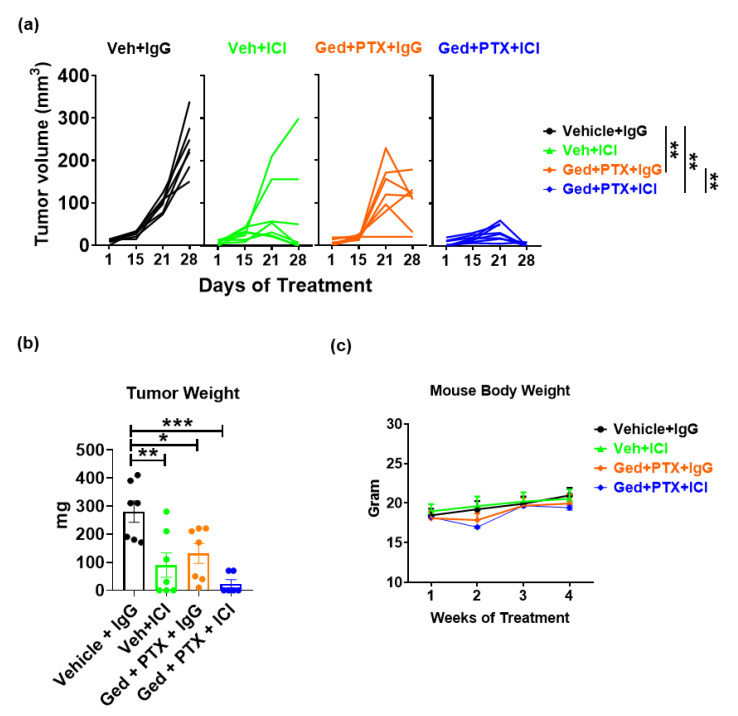

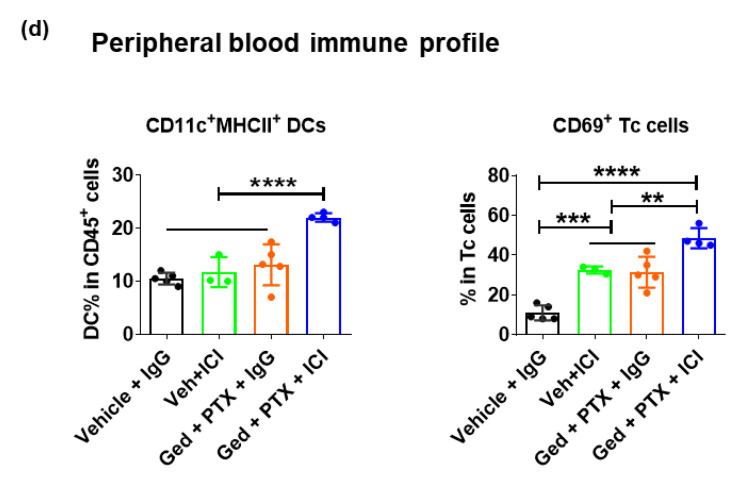
Gedatolisib + PTX + immunotherapy resulted in complete regression of 60% of the PyMT breast tumors. Female C57BL/6 mice were injected with 500,000 PyMT cells in the 4th mammary fat pad. Mice were treated with immune therapy (CTLA4 at 100 ug/mouse and PD-1 Abs at 200 ug/mouse every 3 days, intraperitoneal) and/or gedatolisib (12 mg/kg, twice a week, intravenous), 10 mg/kg PTX (every 3 days, retroorbital), and vehicle plus isotype IgG antibodies were treated as control. Treatments were started 1 week post tumor cell implantation and mouse body weight was monitored. Tumor weights were measured at day 28. Peripheral blood samples at 28 days post-treatment were analyzed by flow cytometry. (**a**–**c**), n ≥ 7 per group; (**d**), n = 3–5 per group. Where indicated, * *p* < 0.05; ** *p* < 0.01; *** *p* < 0.001; and **** *p* < 0.0001.

While the combination treatment of gedatolisib and ICI suppressed PyMT tumor growth, no tumors completely regressed. Recently, gedatolisib was reported to have a tolerable safety profile when combined with PTX in a phase I dose-escalation study with advanced ovarian, endometrial, and non-small cell lung cancers [19]. We further investigated whether the addition of PTX to the gedatolisib treatment might enhance sensitivity to ICI therapy in the PyMT model. After 4 weeks of treatment, there was a significant reduction in tumor volume (Figure 5a) and tumor weight (Figure 5b) in mice treated with vehicle + ICI, gedatolisib + PTX + IgG and gedatolisib + PTX + ICI, compared to vehicle + IgG-treated mice. Impressively, 4/6 tumors from mice treated with gedatolisib + PTX + ICI completely regressed, while the remaining 2/6 tumors exhibited an 85% growth inhibition compared to the vehicle + IgG-treated tumors. Furthermore, gedatolisib + PTX + ICI introduced mild toxicity in the first week of treatment based on an ~10% loss of mouse body weight, which recovered afterwards (Figure 5c), consistent with the tolerable safety profile of gedatolisib + PTX in patients [19].

The regression of tumors in the gedatolisib + PTX + ICI treatment group made it impossible to ascertain the effect of treatment on specific subpopulations of immune cells in the TME. To better understand the anti-tumor immune response to this drug regimen, we immune-profiled the peripheral blood samples after 28 days of treatment in each mouse (Figure 5d). Gedatolisib + PTX + ICI, but not gedatolisib + PTX + IgG or vehicle + ICI, significantly promoted MHCII^+^CD11c^+^ DC abundance compared to vehicle + IgG (*p* < 0.0001), gedatolisib + PTX + IgG (*p* = 0.0008), or vehicle + ICI (*p* = 0.0007). Furthermore, vehicle + ICI (*p* = 0.0006) or gedatolisib + PTX + IgG (*p* = 0.0002) significantly increased the frequency of CD69^+^CD8^+^ activated Tc cells to ~31% compared to 11% in the vehicle + IgG-treated mice. Notably, the addition of gedatolisib + PTX into ICI therapy (49%, *p* = 0.0091) resulted in a further induction of CD69^+^CD8^+^ activated Tc cells in the blood circulation compared to vehicle + ICI. These data show that dual inhibition of PI3K/mTOR plus PTX shifts the immune cell content of tumors to reverse the “cold” immune phenotype and increases sensitivity to ICI therapy.

### 2.6. PIK3CD and PIK3CG Are Preferentially Expressed in the Lymphocytes of Triple-Negative BC Patient Tumors

**Figure 6:**
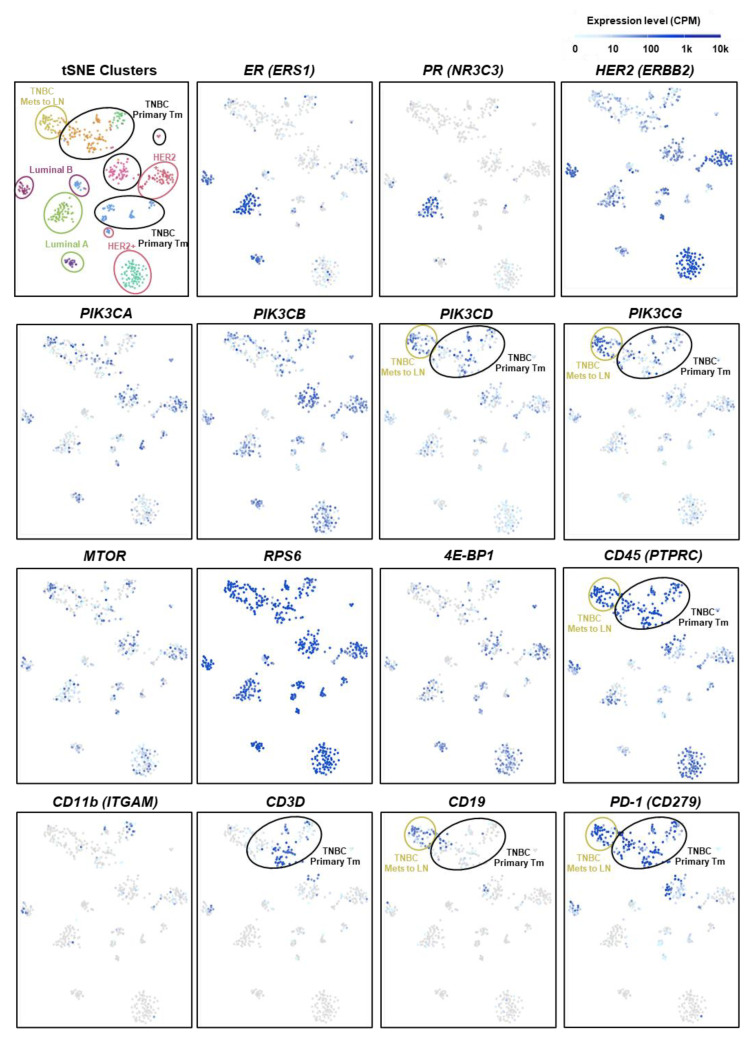
*PIK3CD* and *PIK3CG* are preferentially expressed in the lymphocytes of TNBCs. Results of tSNE and gene expression profile were retrieved from Single Cell Expression Atlas (https://www.ebi.ac.uk/gxa/sc/experiments/E-GEOD-75688/) (accessed on 7 December 2020). Analysis of scRNAseq from breast cancer cells from primary and lymph node metastases from 11 patients representing the four subtypes of breast cancer: luminal A, luminal B, HER2, and triple-negative breast cancer. Number of cells: 539. t-SNE perplexity = 25, color plot by: K = 10.

Molecular analysis with gene expression profiling showed that BC could be sub-classified into different subtypes: luminal ER-positive (luminal A and luminal B), HER2-enriched, and triple-negative or basal-like BCs [20,21]. To explore the relative gene expression of PI3K/mTOR-associated genes in BC subtypes, we analyzed the publicly available single-cell (sc) RNA-seq dataset from Single Cell Expression Atlas (https://www.ebi.ac.uk/gxa/sc/home) (accessed on 7 December 2020) containing 515 cells from 11 BC patients representing the four subtypes (Figure 6). While *PI3KCA* (encoded for PI3Kα) and *PI3KCB* (encoded for PI3Kβ) are generally expressed among all four BC subtypes, *PI3KCD* (encoded for PI3Kδ) and *PIK3CG* (encoded for PI3Kγ) are preferentially expressed in the primary and metastatic TNBC. The mTOR pathway-related genes, including *MTOR*, *RSP6,* and *4E-BP1*, are ubiquitously expressed in BC subtypes. Further t-SNE analyses suggested that the cells that expressed high levels of *PI3KCD* and *PIK3CG* are CD45^+^CD3^+^ T- or CD45^+^CD19^+^ B- lymphocytes, but not CD11b^+^ myeloid cells. PD-1 is expressed on activated CD4^+^ and CD8^+^ T cells and the interaction of PD-1 with its ligands (PD-L1/L2) results in the suppression of T-cell activity [22]. Interestingly, those *PI3KCD*^hi^*PIK3CG*^hi^ lymphocytes also exhibited a high level of *PD-1* mRNA expression, suggesting an early T-cell exhaustion phenotype and highlighting a potential beneficial effect of PI3Kγ/δ inhibition in TNBC.

## 3. Discussion

In this study, we show that it is essential to simultaneously inhibit PI3K isoforms to effectively enhance response to PTX and ICI therapy in the PyMT tumor model. PI3Kα inhibition by alpelisib plus PTX did reduce PyMT tumor volume but did not sensitize the tumors to ICI. Evidenced in both our preclinical animal model and clinical trials, alpelisib + PTX induced beneficial, but not durable, granulocyte anti-tumor responses. As a major cellular component of granulocytes, neutrophils can be polarized and display different phenotypes spreading from an antitumor (N1-like) to a pro-tumor (N2-like) phenotype [23,24]. Recent studies in experimental models have provided extensive support for the existence of pro-angiogenic (N2) or anti-tumor (N1) neutrophil phenotypes [23]. The N1 neutrophils are able to generate reactive oxygen species (ROS) and release various pro-inflammatory proteins from intracellular granules, which include myeloperoxidase, lactoferrin, defensin, lysozyme, and proteases (e.g., elastase and gelatinase). These factors can cause tissue damage and tumor cell lysis, as well as potential DNA damage [25]. However, while the neutrophil-driven innate immune response is fast and generally effective, it lacks specificity and the capacity to build long-lived immunological memory compared to the adaptive immune responses, such as T-cell responses. The preferential induction of T cells by the treatment of PI3K/mTOR inhibitor gedatolisib plus PTX, compared to transiently induced granulocytes by PI3Kα inhibitor plus PTX, may partially explain why gedatolisib + PTX better sensitized the tumors for ICI therapy.

Since PI3Kα is often constitutively activated in breast tumor cells, its inhibition should have inhibited PyMT tumor growth but have limited effects on the immune cells, while PI3Kγ and PI3Kδ are the major PI3Ks expressed in leukocytes. Studies have shown that the inhibition of PI3Kδ reduces myeloid-derived suppressor cells (MDSC) and Tregs [26], and the inhibition of PI3Kγ reduces MDSC and shifts the macrophages in the TME toward an anti-tumor M1-like phenotype [16,27]. Thus, it is not surprising that others have shown that PI3Kα,δ inhibition significantly reduces tumor growth in 4T1, MC-38, and CT-26 tumors by reducing tumor-infiltrating Tregs, MDSCs, and protumor macrophages, while increasing CD8^+^ Tc-cell activation and memory [28]. Treatment with the FDA-approved pan-PI3K inhibitor copanlisib plus PTX also resulted in a significant reduction in PyMT tumor growth. ICI removes inhibitory signals of T-cell activation, which enables tumor-reactive T cells to overcome regulatory mechanisms and mount an effective antitumor response [4,29,30]. When combined with ICI therapy, there was a significant increase in the immune cell infiltration into tumors, with increased activation of CD8^+^ Tc cells.

Our previous and current data suggest that while pan-PI3K inhibition enhances response to ICI therapy in mammary tumors, the limited efficacy does not override the significant toxicity of these inhibitors in the clinic. In contrast, gedatolisib is reasonably well-tolerated and functions essentially as a PI3K/mTOR inhibitor [19]. The mTOR inhibitor everolimus has been previously used to treat breast cancers, but toxicity is a major issue with this drug and many patients stop treatment due to this issue [12]. Here, we utilized a PI3K/mTOR dual inhibitor with reduced toxicity as an alternative way to inhibit multiple forms of PI3K and enhance response to ICI therapy. Our data suggest that treatment with gedatolisib + PTX increases sensitivity to ICI therapy. However, our study has limitations. Firstly, in our animal studies, we utilized only one breast cancer model, the PyMT model, which exhibits constitutive activation of the PI3K pathway [31]. Nevertheless, our findings in the mouse model are supplemented with data analysis from human studies, though the number of patients analyzed was small. Clearly additional trials will be needed. Secondly, we utilized αCTLA4 + αPD1 as the ICI therapy in preclinical studies; future work is needed to evaluate the feasibility of combining gedatolisib + PTX with αPD1 or αCTLA4 alone to potentially reduce the ICI-induced immune-related adverse events. Lastly, IMpassion130 reported that αPD-L1 antibody atezolizumab in combination of nab-paclitaxel significantly improved PFS and overall survival (OS) of patients with unresectable locally advanced or metastatic TNBC [8]. However, the primary results from IMpassion131 suggested that atezolizumab in combination with PTX did not improve PFS or OS versus placebo + PTX in TNBC while the cellular or molecular mechanisms are not clear [32]. In the present study, we showed that PTX monotherapy did not inhibit tumor growth in PyMT preclinical mouse models (Figure 2a). The combination of PTX with PI3K/mTOR inhibitor gedatolisib exhibited a delayed response after 21 days of treatment and the further addition of αCTLA4 + αPD1 advanced the therapeutic response from onsite (Figure 5a). It is intriguing to explore whether the backbone of PTX (nab-paclitaxel versus PTX) and/or the classes of ICI (αPD-L1 versus αPD1 +/− αCTLA4) would impact the therapeutic outcomes. Given that new combinations of the inhibitors of the PI3K/AKT/mTOR signaling pathway could provide a new perspective for the management of breast cancers, especially for the cases with precision medicine and drug resistance, future work is need to explore the therapeutic potential and safety profile of mTORC1 inhibitors (e.g., everolimus, temsirolimus and ridaforolimus), mTORC1/2 inhibitors (e.g., vistusertib), as well as AKT inhibitors (e.g., ipatasertib) monotherapy and in combination with ICI.

Current clinical trials of BC are combining gedatolisib with a range of other therapies, including herzuma (NCT03698383), PTK7-ADC (NCT03243331), palbociclib (NCT02626507 and NCT02684032), fulvestrant and letrozole (NCT02684032), and cisplatin and docetaxel (NCT01920061) (ClinicalTrials.gov, accessed on 7 December 2020). Preliminary results from a recent ongoing trial showed that gedatolisib exhibited antitumor activity and a tolerable safety profile when combined with carboplatin and weekly PTX in the treatment of advanced solid tumors (NCT02069158) [19]. New clinical trials are needed to test the combination of gedatolisib, PTX, and ICI therapies in BC. Given the preferentially high expression levels of *PI3KCD* and *PIK3CG* in TNBC, and the preclinical data from this study, along with the data from the existing clinical trials testing gedatolisib + PTX, we suggest that combined treatment with gedatolisib, PTX, and ICI will lead to a durable therapeutic response for TNBC patients.

## 4. Methods

### 4.1. Patients and Samples

This study was conducted according to the Vanderbilt University Medical Center IRB, approval number 140264 (13 November 2015–13 November 2018). All patient donors signed an informed consent before providing tissue samples. Human granulocytes were isolated by gradient centrifugation from fresh peripheral blood of 8 breast cancer patients who were HR^+^/HER2^+^, HR^+^/HER2^−^, or HR^−^/HER2^−^ enrolled in clinical trials NCT01791478, NCT01872260, or NCT02379247 at Vanderbilt University Medical Center. Detailed trial design and clinical characteristics of the patients enrolled were published previously [15]. Briefly, patients enrolled in NCT01791478 and NCT01872260 Arm 2 were given 250–600 mg (escalating dose) of alpelisib daily for 28 days, and 2.5 mg of letrozole daily for 28 days. Patients enrolled in NCT02379247 were given 250–600 mg (escalating dose) of alpelisib daily and 100 mg/kg of nab-paclitaxel on days 1, 8, and 15 of each 28-day cycle. The granulocyte layer at the interface of Histopaque(H)-1077 (Sigma, St. Louis, MO, USA) and H-1119 (Sigma) were collected by density gradient centrifugation [33]. Efforts were made to collect blood at each cycle of therapy, though in some cases it was not possible to obtain blood at every cycle. For killing assays, 30-fold peripheral blood-sorted granulocytes were co-cultured for 18 h with luciferase reporter-bearing MCF7 or MDA-MB-231 human breast cancer cells (obtained from ATCC, Manassas, VA, USA). Plate-attached tumor cells were lysed in situ and luciferase activity was recorded with GloMax luminescent reader (Promega, Madison, WI, USA).

### 4.2. Mouse Studies

The polyomavirus middle T (PyMT) breast cancer cell line was provided by the Hal Moses laboratory (Vanderbilt, Nashville, TN, USA) and had been previously characterized in our lab [34]. Cells were maintained in Dulbecco’s Modification of Eagle’s Medium (DMEM)-F12 medium in a humidified 5% CO_2_ incubator at 37 °C. Cell media was supplemented with 10% heat-inactivated fetal bovine serum (FBS) and 1% penicillin-streptomycin (P/S) solution, and cultures were tested monthly for mycoplasma (e-MycoTM Plus, LiliF Diagnostics, Gyeonggi-do, Korea). PyMT cells (1 × 10^5^) were injected into the fourth mammary fat pad of 8–10-week-old C57BL/6 mice. Mice were sorted to have similar average starting volume among treatments. Mouse body weight was assessed once a week and tumor measurements were taken twice a week with micro-calipers. Tumor volume was estimated as 0.5 × length × width × width. Treatment began when tumors reached 50–150 mm^3^ volume on average or 1 week post-tumor cell implantation and continued until tumors exceeded 15 mm in diameter or became perforated. Drug dosage and delivery of all drugs are summarized in Supplementary Table S1. Animal studies were approved by the Vanderbilt IACUC under protocol M1600058-01 (17 July 2016–17 July 2019) and M1900098-00 (28 October 2019 to 28 October 2022) and conducted in accordance with IACUC guidelines.

### 4.3. Multicolor Flow Cytometry

To phenotype immune cells in the blood, bone marrow, and tumor, approximately 10^6^ cells were isolated, stained with LIVE/DEAD™ Fixable Aqua Dead Cell Stain Kit (ThermoFisher, Grand Island, NY, USA), and blocked using an anti-mouse CD16/CD32 antibody (BD Biosciences, San Jose, CA, USA) for 20 min (4 °C) and then labeled with monoclonal antibodies (Biolegend, San Diego, CA USA) against markers of interest. The antibodies used in the mouse study include: CD45-APC/Cy7, Ly6G-PE/Cy7, CD11c-BV421, F4/80-APC, CD11b-Alexa488, Ly6C-BV605, MHCII-AlexaFlour700, CD206-PE, CD8-PerCP/Cy5.5, CD103-BV785, CD3-PerCP/eFluor710, CD4-BV421, CD8-AlexaFlour700, NK1.1-FITC, PD-L1-PE, CD69-APC, CD25-PE/Cy7, TIM3-BV605, LAG3-BV785, CD44-BV650, and CD62L-BV570. Cell were washed and resuspended in fixation buffer (PBS containing 1% formalin). A total of up to 200,000 single cell events were collected using a 4-Laser Fortessa and the data were analyzed using FlowJo 10.5.3 software.

### 4.4. Analysis of Publicly Available Dataset

Single cell RNA-seq results of primary breast cancer cells and lymph node metastases from 11 patients representing the four subtypes of breast cancer (luminal A, luminal B, HER2, and triple-negative breast cancer) were obtained from Single Cell Expression Atlas (https://www.ebi.ac.uk/gxa/sc/experiments/E-GEOD-75688/) (accessed on 7 December 2020). [35]. A total of 539 cells were sequenced from individual tumor tissues containing carcinoma and non-carcinoma microenvironment cells. Further t-distributed stochastic neighbor embedding (t-SNE) analyses were used to visualize the results. For all t-SNE plots, perplexity = 25, color plot was generated by cluster number K = 10.

### 4.5. Statistical Analysis

Statistical analyses were performed using R 3.6.0 or GraphPad Prism software version 8.3.0. Data are summarized in figures expressed as mean ± SD. Treatment effects in standard two-group experiments were compared using a two-sample t-test with unequal variances or the Wilcoxon rank-sum test. For more than two-group experiments, a one-way ANOVA with post hoc test was used to compare the differences between treatments. For clinical trial analysis, fold-change was calculated for the change in CD45^+^CD66b^+^ granulocyte population in the peripheral blood from the 8 BC patients with complete cycle 1 data. In addition, the duration of response was calculated for each of the 8 patients, and a correlation analysis was performed on the fold-change of CD45^+^CD66b^+^ granulocyte population in relation to the duration of patient response. The Spearman correlation coefficient or a quadratic polynomial regression was used to evaluate the association between two continuous variables. Where indicated, * *p* < 0.05; ** *p* < 0.01; *** *p* < 0.001; and **** *p* < 0.0001.

## Data Availability

Publicly available datasets were analyzed in this study. This data can be found here: Single Cell Expression Atlas (https://www.ebi.ac.uk/gxa/sc/experiments/E-GEOD-75688/) (accessed on 7 December 2020).

## Supplementary Materials

The following are available online at https://www.mdpi.com/article/10.3390/ijms22105207/s1, Figure S1: Immune profiling of tumors in mice treated with PI3K⍺ isoform specific inhibitor, alpelisib, paclitaxel and immunotherapy, Figure S2: Alpelisib induces beneficial, but not durable, granulocyte responses in breast cancer patients, Figure S3: Gedatolisib + Immune induces systemic responses of DC and CD8+ T cells, Table S1: Drug and dosing information.
