# Supplementary material for: Inhibition of the PI3K/mTOR Pathway in Breast Cancer to Enhance Response to Immune Checkpoint Inhibitors in Breast Cancer
Source: Int J Mol Sci. 2021 May 14;22(10):5207. doi: 10.3390/ijms22105207 (PMC8156389; doi:10.3390/ijms22105207)
Supplement: Supplementary file 1 [file ijms-22-05207-s001.zip › ijms-1209432-supplementary.pdf]

(a)

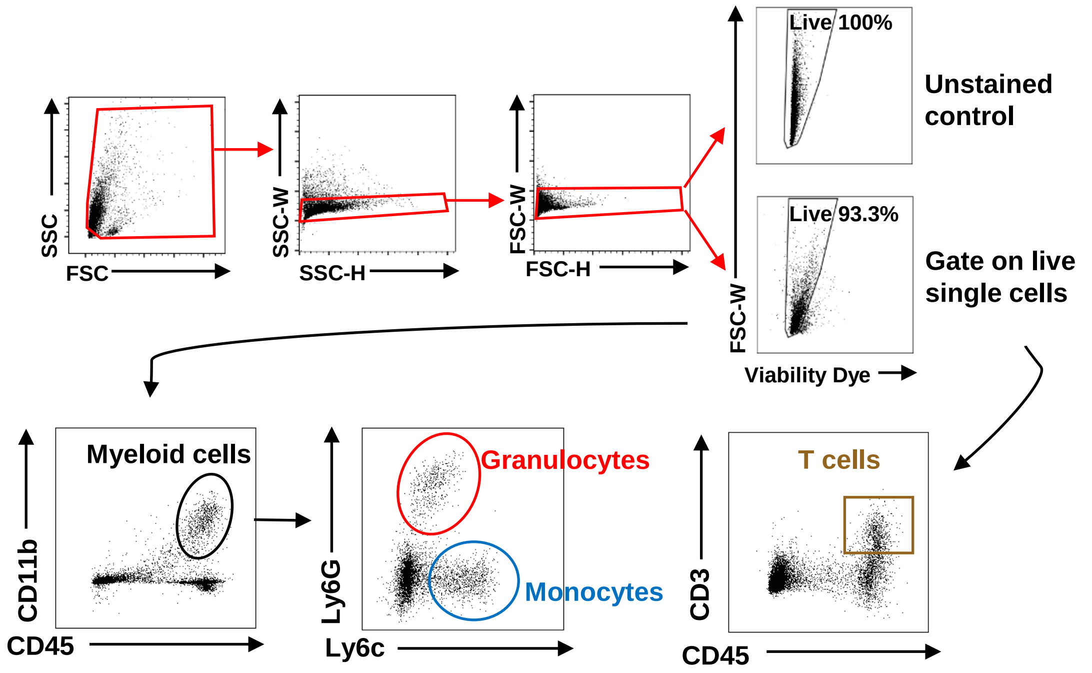

(b)

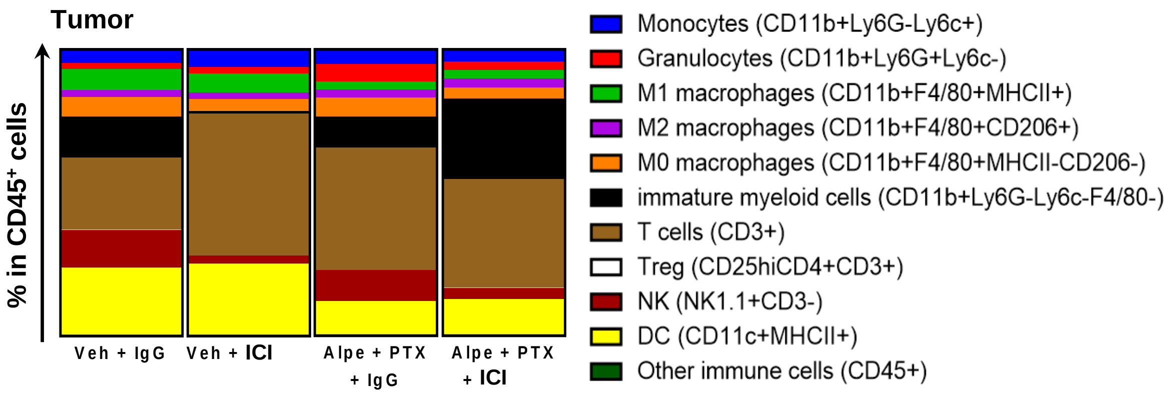

Supplementary Figure S1. Immune profiling of tumors in mice treated with PI3Kα isoform specific inhibitor, alpelisib, paclitaxel and immunotherapy. Female C57BL/6 mice were injected with 500,000 PyMT cells in mammary fat pads, and treatment with 50mg/kg alpelisib (5 days/week), 10mg/kg PTX (every 3 days), and ICI (200ug/mouse anti-PD-1 and 100ug/mouse anti-CTLA-4 every 3 days) was started when tumors reached 125mm^3. Primary PyMT tumor samples were collected after 15 days of treatment and immune profiled by flow cytometric analyses (n=5 per group).

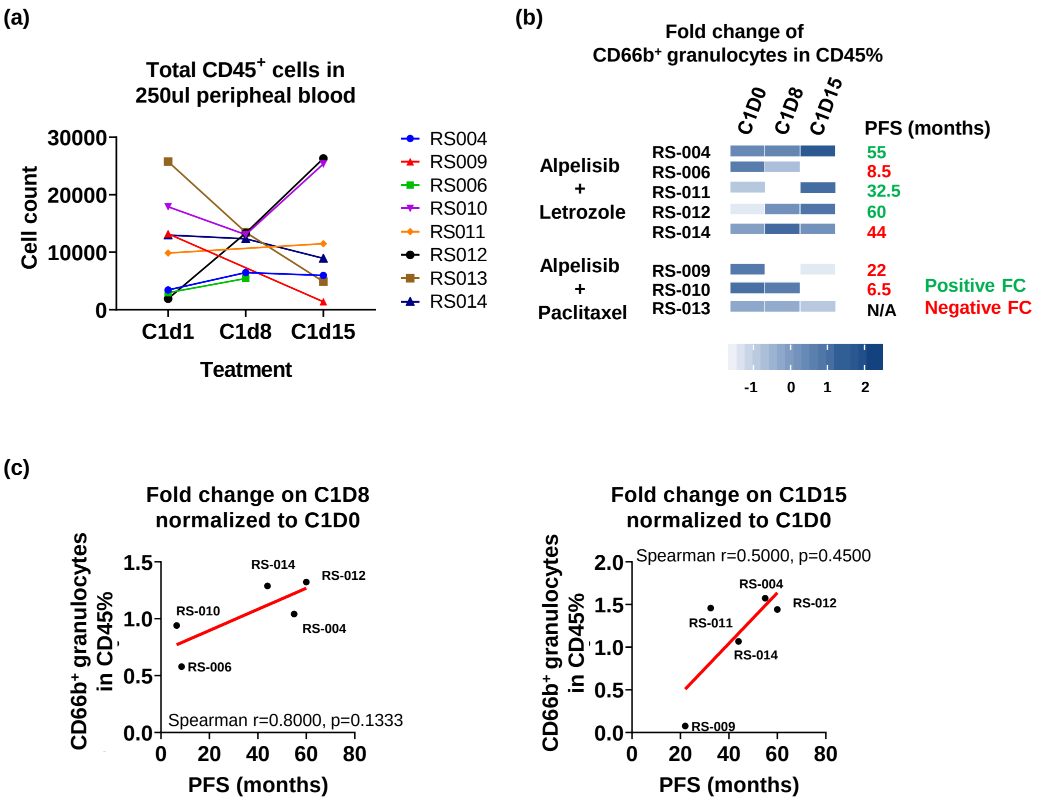

Supplementary Figure S2. Alpelisib induces beneficial, but not durable, granulocyte responses in breast cancer patients. (a) Cell count of total CD45+ leukocytes isolated from 250ul peripheral blood of patients. (b-c) Correlation between CD45/ CD66b granulocytes fold-change during cycle 1 treatment, and duration of therapeutic response in MBC patients enrolled in two clinical trials. FC, fold change. C, cycle of treatment. d, days of treatment in the cycle.

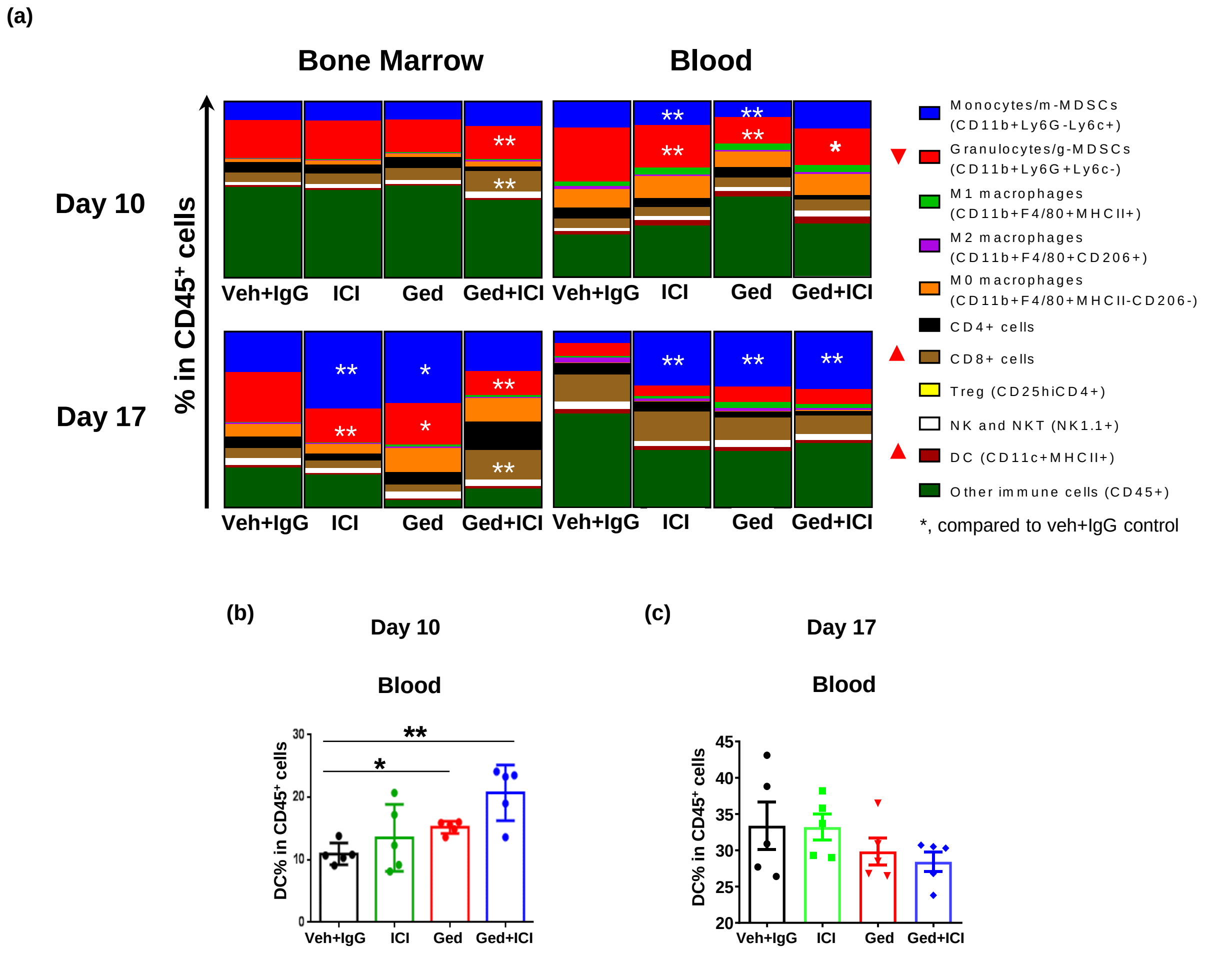

Supplementary Figure S3. Gedatolisib + Immune induces systemic responses of DC and CD8^{+} T cells. Female C57BL/6 mice were injected with 500,000 PyMT cells in fat pad. Mice were treated with immune therapy (CTLA4 at 100 ug/mouse and PD-1 Abs at 200 ug/mouse every 3 days) and/or Gedatolisib (12 mg/kg, twice a week), and vehicle plus isotype IgG antibodies were treated as control. Treatments were started when tumors reached 150mm^3. Immune profiling of bone marrow (BM) and blood samples were analyzed by flow cytometry (n=5 per group).

Supplementary Table S1. Drug and dosing information.

Development I.D.	Name	Target	Dosage and Route of Administration
BYL719	Alpelisib	PI3Kα	50 mg/kg, PO, 5 days a week
BAY80-6946	Copanlisib	pan-PI3K	0.8 mg/kg, IV, every 2 days
PF-05212384/ PKI-587	Gedatolisib	pan-PI3K & mTOR	12 mg/kg, IV, twice weekly
	Paclitaxel	Microtubule stabilizer	10mg/kg, RO, every 3 days
Anti-PD-1	Nivolumab	PD-1 (RMP1-14)	200 ug/mouse, IP, every 3 days
Rat IgG2a	N/A	Isotype control (2A3)	200 ug/mouse, IP, every 3 days
Anti-CTLA-4	Ipilimumab	CTLA-4 (9H10)	100 ug/mouse, IP, every 3 days
Syrian Hamster IgG	N/A	Isotype control	100 ug/mouse, IP, every 3 days

Note: PO, per os or orally. IV, intravenous. IP, intraperitoneal. RO, retroorbital.
